# Investigating the Postprandial Metabolome after Challenge Tests to Assess Metabolic Flexibility and Dysregulations Associated with Cardiometabolic Diseases

**DOI:** 10.3390/nu14030472

**Published:** 2022-01-21

**Authors:** Gaïa Lépine, Marie Tremblay-Franco, Sabrine Bouder, Laurianne Dimina, Hélène Fouillet, François Mariotti, Sergio Polakof

**Affiliations:** 1 Université Clermont Auvergne, INRAE, UMR 1019, Unité Nutrition Humaine, 63000 Clermont-Ferrand, France; gaia.lepine@inrae.fr (G.L.); sabrine.bouder@outlook.fr (S.B.); laurianne.dimina@agroparistech.fr (L.D.); 2 Université Paris-Saclay, AgroParisTech, INRAE, UMR PNCA, 75005 Paris, France; Helene.Fouillet@agroparistech.fr (H.F.); mariotti@agroparistech.fr (F.M.); 3 Toxalim (Research Centre in Food Toxicology), Université de Toulouse, 31300 Toulouse, France; marie.tremblay-franco@inrae.fr; 4 Axiom Platform, MetaToul-MetaboHUB, National Infrastructure for Metabolomics and Fluxomics, 31300 Toulouse, France

**Keywords:** challenge meal, insulin resistance, nutritional phenotypic flexibility, nutrition, omics, oral glucose tolerance test (OGTT), oral lipid tolerance test (OLTT), postprandial physiology, type 2 diabetes

## Abstract

This review focuses on the added value provided by a research strategy applying metabolomics analyses to assess phenotypic flexibility in response to different nutritional challenge tests in the framework of metabolic clinical studies. We discuss findings related to the Oral Glucose Tolerance Test (OGTT) and to mixed meals with varying fat contents and food matrix complexities. Overall, the use of challenge tests combined with metabolomics revealed subtle metabolic dysregulations exacerbated during the postprandial period when comparing healthy and at cardiometabolic risk subjects. In healthy subjects, consistent postprandial metabolic shifts driven by insulin action were reported (e.g., a switch from lipid to glucose oxidation for energy fueling) with similarities between OGTT and mixed meals, especially during the first hours following meal ingestion while differences appeared in a wider timeframe. In populations with expected reduced phenotypic flexibility, often associated with increased cardiometabolic risk, a blunted response on most key postprandial pathways was reported. We also discuss the most suitable statistical tools to analyze the dynamic alterations of the postprandial metabolome while accounting for complexity in study designs and data structure. Overall, the in-depth characterization of the postprandial metabolism and associated phenotypic flexibility appears highly promising for a better understanding of the onset of cardiometabolic diseases.

## 1. Introduction

This review focuses on how metabolomics can improve the evaluation of the metabolic response in humans during nutritional challenges and can reveal subtle changes related to the early onset of metabolic diseases. Although this review is not intended to be exhaustive, it covers most of the commonly applied challenge tests, including single nutrient tests (such as the oral glucose tolerance test (OGTT)), mixed liquid meals (most of them rich in fat, sugar and energy) and complex meals (healthy or not). 

Targeted populations include subjects with different cardiometabolic statuses, ranging from clinically healthy to increased risk factors (obesity, insulin resistance and hyperlipidemia) or overt diseases, such as type 2 diabetes (T2D) and cardiovascular diseases.

## 2. The Postprandial Metabolism as a Window to Explore Phenotypic Flexibility

Feeding, one of the major physiological needs for living organisms, involves a dynamic transition from a fasted state to a postprandial period where ingested food is sequentially digested, absorbed and further metabolized. This postprandial phase represents a major change for the individual’s physiology, given that the body has to pass through the fasting-to-fed transition and handle the arrival of nutrients and energy. 

Historically, the metabolic changes induced by ingestion were studied from the energetic viewpoint (according to *specific dynamic actions*) after which complex changes in metabolism were studied extensively in order to identify the underlying mechanisms and the various determinants responsible for the variation in the magnitude and duration of this metabolic response [1]. As reviewed by Secor, the magnitude and duration of the postprandial metabolic response is dependent upon the features of the meal, the characteristics of the individual and the environmental conditions [1]. 

During fasting, metabolism functions at steady state to ensure physiological functions with consistency; however, this requires regular food intake to compensate for nutrient losses. Conversely, the postprandial phase is associated with an influx of nutrients that must be handled by the organism while maintaining circulating concentrations within acceptable ranges. Thus, the dynamic follow-up of the postprandial phase relates to the capacity of the individual to handle the metabolic disturbances associated with the switch from the fasted to fed state according to “hemodynamic” type processes [2]. Therefore, the intrinsic capacities of an individual to handle a given meal or nutritional test are especially interesting as they provide additional information on the efficacity of the regulation systems in play, which are not visible in the steady post-absorptive state. In addition, by standardizing the composition of the ingested meal, individual responses can be compared, including between individuals with different pathophysiological statuses.

Studies on nutrition are limited regarding the establishment of direct relationships between nutrients, foods or diets and health. This is because changes induced by diet are often long and subtle and also because human physiology can respond flexibly to changes in the environment, including changes related to what we eat. This capacity of response, resulting in the short-term maintenance of homeostasis or metabolic resilience, also called *phenotypic flexibility*, has opened the door to a new approach to exploring health status and its slow and subtle progress to disease onset [3]. 

In this context, conducting postprandial nutritional studies appears to be an excellent strategy to further characterize this *nutritional phenotypic flexibility* as a central characteristic of health (rather than a disease symptom) [4]. Indeed, in healthy conditions, the human body elicits a complex metabolic response, consisting of the reorganization of metabolic processes, which results in managing meal nutrients efficiently while avoiding excessive metabolic shifts that would challenge homeostasis. However, this regular nutritional function may be impaired, and nutritional metabolism may display a certain degree of inflexibility. 

Recent studies have led to the identification of key mediators that regulate the physiological processing of meal nutrients and explain how short-term metabolic effects contribute to disease. Clinical and preclinical studies have uncovered mechanisms by which elevated postprandial concentrations of nutrients (e.g., glucose) promote vascular dysfunction via oxidative-stress-related pathways [5]. This research may well explain how meal metabolism, when repeated daily throughout a lifetime, contributes to the long-term initiation and development of cardiometabolic diseases [6]. 

Considering that the human body functions in a postprandial state for most of the day [7], the capacity to handle meals appears to be one of the contributing factors to the increasing prevalence of obesity and cardiometabolic disease in Western societies [8,9,10]. Since the original definition of health status by the World Health Organization in 1948 [11], defining the health status of an individual has evolved towards a more holistic and dynamic description [12], including, among others, the capacity to adapt to the environment and to respond to a new variety of social, physical and emotional challenges with resiliency. As with many other “components” of the environment, individuals are exposed to what they eat. Nutrition can therefore represent another source of challenge to which individuals must adapt. 

Given the importance of maintaining this nutritional phenotypic flexibility as a key feature of optimal health, the objective of characterizing flexibility and identifying nutritional stress-derived (health) biomarkers has been developed [3,13]. Nutritional challenge tests were specifically designed to assess this flexibility and thus reveal the subtle physiological changes that are not visible in the static (fasting) state [14,15]. Analyzing the dynamic response to a given nutritional challenge test therefore makes it possible to assess the degree of flexibility and thus detect the earliest signs of reduced flexibility (or dysmetabolism) of processes that are regulatory and adaptive in order to maintain equilibrium between core metabolic processes. This concept evolved further with the notion of Nutritional Biomarkers of Health, which moves on from the challenge test to the postprandial food evaluation, combining the nutritional and medical sciences into a single strategy [4].

In recent years, a large number of studies have been conducted with the objective of assessing phenotypic flexibility. The concept was well illustrated in the study performed by Kardinaal et al. in clinically healthy and metabolic syndrome volunteers. Both groups underwent a high fat test aiming at challenging the postprandial metabolism, and the response of several biomarkers related to metabolism, inflammation and vascular endothelial dysfunction were measured [16]. In the healthy group only, the test was carried out after 4 weeks of consumption of a hypercaloric diet, which was expected to intensify the adaptive and regulatory mechanisms aimed at maintaining the homeostatic control of the main metabolic processes. The major core processes (glucose, triglycerides and inflammation metabolites) were not altered in these subjects; however, several markers, such as adipokines, essential fatty acids, endothelial adhesion molecules and stress-related markers, were induced during the postprandial (high fat) phase, revealing that overfeeding had induced metabolic stress and driven processes. In contrast, these regulatory processes were not seen in the metabolic syndrome subjects, who instead showed increased postprandial levels in several markers as well as a blunted metabolic stress response. Overall, this showed that, upon prolonged caloric overload, these adaptive processes may reach their limits, and compensatory mechanisms may cause the damages resulting from the derailment of core metabolic processes, resulting in insulin resistance (IR), plaque formation and low-grade inflammation.

## 3. Metabolomics, Nutrition and Challenge Tests

In its recent transition from a reductionist strategy (targeted nutritional biochemical approaches) to a more integrative, holistic and comprehensive strategy (non-targeted whole metabolome approaches), nutrition research is evolving into a model in which metabolomics has a central role in providing a complete description of the physiological and biochemical responses to a given dietary intervention [17]. As metabolomics is fundamentally phenotype-driven, *nutrimetabolomics* (metabolomics applied to human nutritional studies) provides better and more individualized biomarkers than classical biochemistry techniques and is expected to give better indicators of dietary effects on a target population [18]. 

Thus, by comparing global metabolic profiles in response to a nutritional change (nutrient, diet and dietary habits) without a priori hypotheses, novel biomarkers of metabolic adaptations or shifts to a new situation can be obtained. Although metabolomics relies mainly on LC-MS (Liquid Chromatography–Mass Spectrometry), GC-MS (Gas Chromatography–Mass Spectrometry)and NMR (Nuclear Magnetic Resonance), no single analytical technique has been able thus far to simultaneously assess all the metabolites in a given sample: a multi-platform analytical strategy currently remains the best strategy to cover as much as possible the complexity of the human metabolome [19], including more than 114,215 currently identified molecules in human tissues [20]. 

Furthermore, studying the postprandial response of the metabolome is complex, often involving cross-over designs and the integration of very large repeated measure datasets and calls for the development of new statistical methods that are able to break down challenge and time effects as well as intra-subject variability [15,21,22]. 

As mentioned above, the integrative and wide-range coverage of the biological processes of metabolomics makes it highly suitable for the exploration of metabolic changes following a nutritional challenge, in particular, to discriminate healthy from pre-disease individuals [23]. Thus, the response of the metabolism to these challenges is reflected in the metabolome as changes in metabolite concentration that can be associated with specific physiological processes responsible for maintaining or losing homeostasis during the healthy-to-disease transition [24]. In a previous compilation mainly focused on glucose homeostasis and challenge tests related to metabolomics, Brennan pointed out that the application of metabolomics analyses to several different challenge tests allowed revealing different responses between healthy subjects with or without IR, which could help to identify pre-disease phenotypes [25]. Similarly, Wopereis et al. [26] also showed that dietary glucose and lipid challenges did not induce a strong acute inflammatory response in healthy subjects, as quantified by an accurate and broad panel of classical parameters. Those findings highlight the need to perform wider and more integrative analyses, such as those provided by metabolomics, as recently discussed by LaBarre et al. [27]. In this sense, combining the more classical homeostatic biomarkers of metabolic perturbations with robust and validated metabolomics-derived biomarkers measured during a nutritional challenge is a promising next step towards improved assessments in nutritional studies [17].

One of the pioneering studies on the application of metabolomics to nutritional challenge tests was conducted by Krug et al. [28], in which 15 young healthy male volunteers underwent several different challenge tests: some of them were physical tests (cold stress and physical exercise) and others were nutritional tests, such as fasting, OGTT, a standard liquid diet and the lipid tolerance test. The authors recalled that the response of the organism presented overall an “accordion like-effect” in the metabolic profiles assessed, which were able to fluctuate as a function of the nature of the challenge applied. The key aspect of this particular study was that, despite a differential metabolic response to the varied challenges, all the subjects presented equivalent metabotypes in basal conditions, which confirmed the suitability of this strategy to reveal more sensitive changes between subjects, depending on their underlying physiological traits. 

In the following sections, we discuss the results from challenge meal studies using a metabolomics approach. Study selection was based on several criteria: we included only clinical studies conducted on adults or adolescents with different cardiometabolic health statuses. Thus, studies recruiting subjects who were clinically healthy or presenting increased risk factors, such as high BMI, IR or dyslipidemia, were selected. We also included studies evaluating the metabolism of subjects with T2D or cardiovascular diseases. Only metabolomics studies on plasma or serum were considered, given the low representativity in the literature of other biological fluids (saliva, urine…). Concerning the meal tests, we only selected studies in which a postprandial response to challenge tests was used as a way to assess health status and phenotypic flexibility in different conditions (in groups with different health statuses or following an intervention). Studies comparing the postprandial responses to specific foods or dietary patterns with the aim of identifying food intake biomarkers were not included. All types of challenge tests were considered if at least two data points were explored (before and after the challenge test). Regarding analytical aspects, untargeted and targeted metabolomics were included without discrimination regarding the analytical method used (NMR, LC-MS or GC-MS). However, we excluded studies with a targeted approach based on a very small and specific subset of metabolites.

## 4. Oral Glucose Tolerance Tests

Due to the traditional glucocentric view of metabolism in men, the OGTT was the first and remains the most widely used challenge test. Since its application to pregnant women in 1957 [29], it also became the main tool for evaluating the flexibility of the glucose regulatory system, both dynamically and in an easy-to-apply way in the framework of large-scale design in order to determine the degree of glucose tolerance and T2D diagnosis.

One of the first studies combining OGTT and metabolomics was conducted by Wopereis et al. [30] in overweight human volunteers during a 9-day intervention with a mild-acting anti-inflammatory drug. The authors demonstrated that the statistical power for detecting the treatment-induced effects was better after OGTT compared to the fasting state. Furthermore, the subtle homeostatic alterations induced by the treatment were only visible by combining the challenge test (OGTT) with the metabolic profiling, thus highlighting the interest of this research strategy.

### 4.1. OGTT in Healthy Individuals

**Figure 1:**
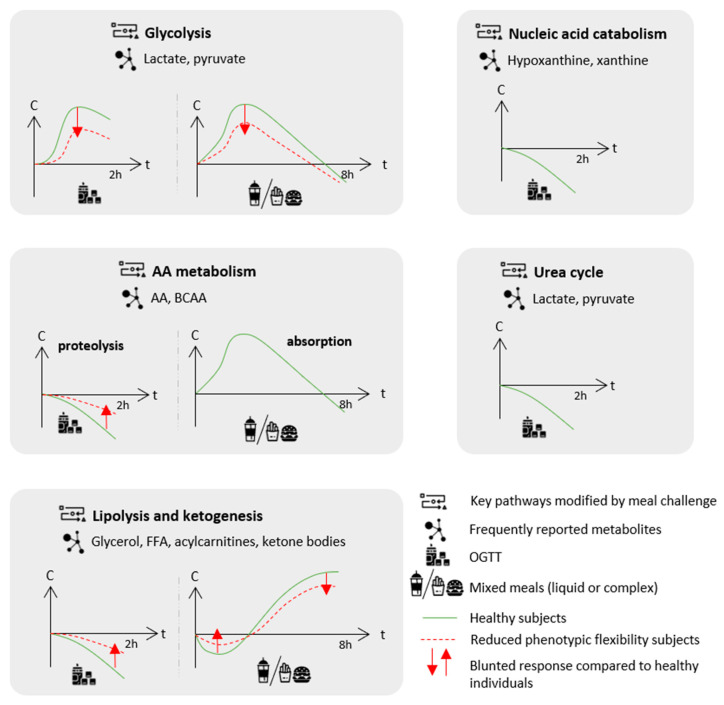
Frequently reported effects of challenge meals on postprandial key metabolic pathways as identified by metabolomics approaches and blunted responses characteristic of a reduced phenotypic flexibility in populations with increased cardiometabolic risk compared to healthy individuals. The metabolite response to the challenge meal is presented as the evolution of concentration (C) over time (t). For details on the metabolites and experimental designs, see Table 1. AA: amino acids; BCAA: branched-chain amino acid; FFA: free fatty acids; and OGTT: oral glucose tolerance test.

The first studies to apply metabolic profiling to investigate the kinetics of human plasma biochemical response to an OGTT in healthy volunteers were conducted in the later 2000s [31,32,33] in small controlled trials. More recently, studies have investigated the metabolomics signatures associated with OGTT in much broader populations [34,35,36,37,38] (see Table 1). Most of the dynamics observed in the metabolome revealed insulin’s known actions along four key directions, reflecting a switch from catabolism to anabolism: proteolysis, lipolysis, ketogenesis and glycolysis (Figure 1). 

First, the inhibition of proteolysis decreased the circulating levels of several amino acids (AA) and, in particular, branched-chain amino acids (BCAA) and aromatic amino acids (AAA) as consistently reported across studies [32,33,34,35,37,38]. Secondly, the switch from the fasted to fed state inhibited lipolysis and reduced the circulating levels of related metabolites, such as glycerol, free fatty acids (FFA) and acylcarnitine (from 60% to 70%) [31,32,33,35,36,38]. Given that acylcarnitines represent by-products of mitochondrial β-oxidation, their reduced levels following the glucose load reflect the switch from fatty acid β-oxidation to glycolysis, as glucose mimics the fed state and becomes the major available energy substrate. 

Zhao et al. also observed that the glucose load exerted a larger and specific reduction of C16:1 and C18:1 circulating acylcarnitines compared with their saturated counterparts, which could be related to the more efficient suppression of lipolysis of mono-unsaturated fatty acid (MUFA)-containing triglycerides (TG) [31]. This profile contrasted with the relatively stable levels of plasma poly-unsaturated fatty acids (PUFA), due to the fact that phospholipase A2 is not inhibited by insulin following the glucose challenge. A recent lipidomics analysis conducted on healthy young adults (*n* = 246) further reported profound changes in the lipidome following an OGTT [36]. Concordant decreased acylcarnitine levels (especially C14:2 with a 73% decrease) were observed as well as decreased levels of many other lipid species, including sphingolipids, sphingomyelins, lysophosphatidycholines, lysophosphatidylethanolamines, lysophosphatidylinositols, diacylglycerols and TG with MUFA and saturated fatty acids (SFA). Thirdly, the OGTT inhibited ketogenesis and was consistently associated with a reduction in circulating β-hydroxybutyrate, the main ketone body [32,34,35,37,46]. Fourthly, glycolysis was stimulated, leading to increased by-product (lactate and pyruvate) levels [32,34,35,37]. Decreased lactate levels were reported only once in healthy subjects [46]. 

Interestingly, the pattern of change of the post-glucose load alterations of those four pathways did not appear to be the same. Glycolysis-related metabolites peaked within 2 h, whilst metabolites related to lipolysis, ketogenesis and proteolysis continuously decreased during 2 h, with a tendency for lipolysis and ketogenesis to be affected faster than the proteolysis products [32,35]. 

Among the metabolites whose concentrations were affected by the glucose bolus, some provided information on unexpected pathways not directly related to glucose homeostasis. Thus, bile acid concentrations showed a dramatic increase following glucose ingestion [31,32,34], which appeared to be related to the capacity of glucose to induce cholecystokinin release and then gallbladder contraction [47]. On the other hand, urea cycle intermediates, such as citrulline and ornithine, were decreased [32,33,34,38], which might indicate decreased gluconeogenesis following glucose ingestion as this metabolic pathway is coupled to urea synthesis [48]. Hypoxanthine and xanthine, whose levels were also decreased [32,34,38], are nucleotide degradation products [49], and their decrease could reflect a switch from the catabolism to anabolism of nucleic acids induced by insulin [50]. TCA cycle intermediates were also affected by the glucose load, either decreased [34,46] or increased [35], and the biological interpretation of these findings remains to be established. Other unexpected pathways included vitamin B (B1, B3, B3, B5 and B6) and serotonin metabolisms [34]. While, in the first case, the reduced levels of B-type vitamins appeared to be related to a glycolysis-driven impact on thiamine, the lower serotonin levels after the glucose challenge could certainly have been related to the tryptophan metabolism.

### 4.2. OGTT in Insulin Resistance, Overweight and Hyperlipidemic Conditions

As explained above, the OGTT is the first and simplest challenge test initially developed to unravel and diagnose glucose intolerance and diabetes when the fasting blood glucose test is not selective enough. Therefore, several studies were designed to explore responses to glucose load more broadly as well as the association of the dynamic changes of the metabolome with health outcomes related to glucose homeostasis. Thus, in several studies, the metabolome response observed in healthy subjects was compared to that observed in IR, overweight or obese and hyperlipidemic patients. 

The specific differences in the postprandial metabolome following an OGTT depending on IR status have been investigated in many studies [32,34,35,36,38,39,40] (detailed in Table 1). IR status was assessed with either fasting plasma insulin [32], glucose, homeostasis model assessment (HOMA)-IR or insulin area under the curve (AUC) following an OGTT [34,36,38] or using a hyperinsulinemic-euglycemic clamp [39]. In the largest scale study, including a total of 5340 Finnish individuals from two independent cohorts, IR classification was based on a combination of fasting insulin levels and OGTT response [35]. 

This pivotal study comparing normal glucose tolerant individuals, either IR or insulin sensitive, demonstrated a blunted response following on OGTT on the four key pathways previously discussed, a finding that was replicated across most studies (Figure 1 and Table 1). IR was associated with blunted glycolysis stimulation, as indicated by smaller increases in lactate and pyruvate [32,34,35]. The inhibition of proteolysis and ketogenesis induced by a glucose bolus was also blunted in IR subjects, as evidenced by smaller decreases in AA (specifically BCAA) and ketone bodies [32,34,35]. A smaller glycerol decrease was also reported, suggesting blunted lipolysis inhibition in association with IR [32,35]. In another study conducted on 470 subjects, the trajectories of nine metabolites (out of the 192 detected), including MUFA, lysophosphatidylethanolamines and medium-chain acylcarnitines C10 and C12, were associated with IR and showed blunted decreases in this condition [39]. The strongest associations were observed for C10 and C12 acylcarnitines. In their lipidomics analysis, Beyene et al. reported wide associations of plasma lipidome with IR and also demonstrated a strong association of C12:0 and C13:0 acylcarnitines with insulin AUC, suggesting the implication of medium-chain acylcarnitines in the IR condition [36]. Finally, the increase in circulating bile acid concentration following OGTT, unexpectedly reported in healthy subjects, was blunted in IR [32,34,39]. 

When comparing T2D and normal glucose tolerant subjects, a recent study reported enhanced differences in metabolomic profiles after the challenge test as only one metabolite was significantly different in the fasted state, while 22 were different 2 h after OGTT [38]. Among these 22 metabolites, 7 were also higher in the T2D compared to pre-diabetic subjects. In their study, Wang et al. compared IR individuals without glucose intolerance with prediabetic and diabetic subjects and showed that the post-load metabolic dysregulations induced in the different groups mostly followed the same trends and were of similar amplitude [35]. Both findings highlighted the interest of a multi-metabolite approach to characterize postprandial dysfunctions and identify individuals at cardiometabolic risk, especially in the early stage of IR. In addition, Shaham et al. evidenced the capacity of a glycerol and leucine/isoleucine model to predict fasting insulin levels, with both metabolites offering complementary and significant explanatory power and, thus, further underlining the interest of more systemic approaches to uncover the multidimensional aspects of the postprandial response [32]. 

Some studies also attempted to compare plasma and serum metabolomes following glucose ingestion in lean and overweight or obese populations [37,40,41,42,43,44] (Table 1). Interestingly, they reported alterations in metabolite postprandial trajectories similar to those observed in IR subjects with noticeably blunted decreases of circulating AA and lipids. While Liu et al. reported a blunted decrease of most AA in obese compared to lean subjects [42], another study reported an opposite trend in obese compared to control subjects with increased levels of several AA (leucine/isoleucine, tyrosine, glutamate and asparagine) 30 min after glucose bolus ingestion [41]. However, the obese subjects presented a more heterogeneous BCAA response than the lean subjects and a comparatively blunted increase for other AA (phenylalanine, serine and aspartate). In addition, both studies reported a blunted FFA response in obese subjects. Labarre et al. also reported a blunted decrease in FFA but also in acylcarnitine and FA oxidation product levels in obese compared to lean adolescents [44]. In addition, in obese or overweight females, but not in males, this blunted acylcarnitine decrease (represented by a postprandial fold change) was positively associated with HOMA-IR. 

Rämö et al. compared the metabolome following an OGTT in monozygotic twins (sharing the same genetic background) with discordant BMI and liver fat content, thus, aiming at deciphering the genetic and environment interaction [37]. Their results were concordant with previous findings as they reported blunted decreases in isoleucine, SFA, MUFA, TG, small very low density lipoprotein (VLDL) and small low density lipoprotein (LDL) in the groups of individuals with high body mass index (BMI) and high liver fat. Interestingly, the dynamic metabolomics profiles of cotwins with discordant BMI but concordant liver fat content were quite similar, thereby, highlighting the importance of the adiposity phenotype rather than the genotype of this particular pathophysiological feature. 

A study investigating the effects of a weight-loss intervention, followed by a weight-maintenance phase conducted in morbidly obese subjects, compared the OGTT postprandial metabolome at the different intervention steps with the metabolomics profiles of lean individuals [43].Overall, the weight-loss program led to the improvement of the postprandial trajectories, e.g., closer to those observed in healthy subjects, for AAA, BCAA, FFA and glycerol, which recovered a greater postprandial decrease after the intervention. For other metabolites, such as β-hydroxybutyrate, the trajectories remained different from those of lean subjects throughout the course of the intervention with blunted decreases. This study also showed that the BCAA and FFA responses to the OGTT were much more heterogenous at baseline then at the end of the weight-loss program. Conversely, Geidenstam et al. reported, in a previous analysis, much higher heterogeneity for BCAA trajectories in obese compared to lean subjects while the FFA response was quite homogenous within both groups [41].

Finally, a few studies investigated the OGTT response of hyperlipidemic subjects, as defined by high TG and high total cholesterol levels [45,46]. Li et al. reported increased postprandial levels, rather than blunted decreases, for many AA in the hyperlipidemic group compared to the healthy group [45] in accordance with the results of Geidenstam et al. in obese individuals cited above [41]. A very significant change was also observed regarding γ-aminobutyric acid (GABA) levels, which increased by 79% in the hyperlipidemic group, whereas it decreased by 38% in the healthy individuals. Interestingly, both GABA and isoleucine postprandial changes were associated with HOMA-IR. In addition, lipid-related endpoints (TG, total cholesterol, LDL and HDL cholesterol) were associated with BCAA and other AA, such as tyrosine and serine. This latter observation supports the idea that the BCAA metabolism interacts with both the glucose and lipid metabolisms [51] given that they are not only associated with the major changes directly related to glucose homeostasis and IR during the OGTT [52,53,54] but can also be modulated by lipid dysregulations. More recently, a second study reported significant differences in the TCA cycle intermediate postprandial trajectories with strong increases in hyperlipidemia compared to healthy subjects [46]. 

## 5. Mixed Challenge Meals

Although the OGTT has been shown to effectively reveal changes in the metabolome and thus discriminate individuals at different levels of cardiometabolic risk from healthy individuals, this test is composed of a single nutrient (glucose) eliciting specific responses. Therefore, other meal tests able to vehiculate several nutrients in a single bolus were designed [14]. Using a metabolomics approach, liquid mixed meals containing all the macronutrients were used to challenge the metabolism during the postprandial period [55,56,57,58,59,60,61,62,63,64]. 

Compared to OGTT, mixed meals induce further hypertriglyceridemia and hyperglycemia [65] and trigger the entire metabolism. Although liquid mixed meals have a more complex macronutrient composition than the OGTT, they remain relatively simple compared to an every-day meal since they result from the simple addition of purified ingredients eliciting no or a very poor food matrix effect. The use of complex challenge meals, including whole foodstuffs and their associated food matrixes, combined with metabolomics analyses have also been reported [66,67,68,69,70,71,72,73]. Given the known impact on digestion and further metabolization of the food matrix and nutrient interactions, postprandial changes following a complex meal are expected to reveal metabolic alterations different from simple glucose boluses or HF meal tests. They also elicit a postprandial metabolism closer to that observed in real life settings.

These different multi-nutrient challenge meals have theoretical advantages over a simple OGTT; however, their heterogeneous composition is their main drawback, as this makes it difficult to generalize the findings obtained with a specific challenge meal to another one as discussed by LaBarre et al. [27]. Macronutrient distribution is either balanced with proportions resembling those observed in a regular diet or tends towards a high contribution of one of them, often lipids or occasionally carbohydrates. Those high-fat (HF) challenge meals further induce low-grade inflammation and endothelial dysfunction [16] and bring additional information compared to balanced meals. The overall energy intake is also contrasted. The caloric-dose effects on the postprandial response were investigated by Bütikofer et al. in healthy normal weight and overweight men following a high-fat complex meal given at three increasing doses (500, 1000 and 1500 kcal) [74]. During the 6-h LC-MS metabolomics follow-up, 1024 features were changed in the fed state, and 135 were caloric-dose dependent. Most metabolites were different between the 500 kcal and both the 1000 kcal and 1500 kcal meals, with the last two eliciting similar responses. This finding emphasized the importance of the overall caloric intake of the challenge meals studied, in particular when aiming at identifying different degrees of metabolic flexibility and thus confirming earlier reports [75,76]. 

The complexity of the food matrix is also very heterogeneous—ranging from the simple addition of liquid fat, sugar and protein extracts to full-fledged meals, including processed food, such as fast-food meals. In this section, we discuss studies related to liquid and complex meals either with balanced macronutrient composition or high lipid or carbohydrate contents. All the different types of challenge meals are included under the designation of mixed meals. Detailed meal compositions, study design description and key results of the studies discussed are given in Table 2 and Table 3. 

To address the considerable heterogeneity in challenge meals used to assess phenotypic flexibility, a recent systematic review aimed at designing a standardized meal challenge specifically designed to trigger most of the regulatory processes involved in homeostasis regulation [14]. The resulting optimal macronutrient combination proposed by the authors, called the PhenFlex drink, was a 950 kcal blend composed of 75 g of glucose (carbohydrates, 33%E), 60 g of palm oil (lipid, 59%E) and 20 g of milk protein concentrate (protein, 8%E). 

Overall, the use of mixed challenge meals was not only able to highlight additional differences within the metabolome that were not visible in the fasted state when comparing healthy controls and subjects with overt disease (T2D) or increased cardiometabolic risk [56,58,63] but also within clinically healthy subjects, thus revealing subtle metabolic dysregulations visible only during the postprandial period [61,62]. For instance, using the PhenFlex drink, Van den Broek et al. classified 100 clinically healthy individuals into different health level groups with contrasted phenotypic flexibility (optimal vs. suboptimal) using “health space” visualization [61]. Young subjects with a low to normal fat percentage had a markedly different position in the health space compared to older subjects with a normal to high fat percentage in all four health domains evaluated: glucose metabolism, lipid metabolism, AA and vitamins and metabolic stress. This was also confirmed when assessing a subset of clinical markers that all showed significantly different values between those two groups. Postprandial follow-up has also been reported to reveal additional differences between groups following nutritional intervention that were not visible in the fasted state [71,72].

### 5.1. Main Metabolic Shifts Induced by Mixed Meals in Healthy Individuals

As explained above, mixed challenge meals trigger a more complex postprandial response than does a single nutrient test (OGTT, OLTT etc.). However, despite their heterogeneous compositions, consistent postprandial metabolic shifts were reported across metabolomics studies in healthy individuals with striking similarities to those elicited by the OGTT, especially during the first hours following meal ingestion (Table 2, Figure 1). 

Wopereis et al. measured the postprandial response of 20 healthy men to the PhenFlex challenge for 8 h with a set of 132 features and reported that 110 changed significantly after a meal, thereby, revealing the ability of the challenge meal to trigger all key homeostatic processes [63]. Glycolysis rapidly increased with pyruvate reaching a peak within 1 h before slowly decreasing—similar to the OGTT post-load response. AA similarly followed a typical absorption curve with a fast increase followed by a return to baseline, in contrast with the decreased AA levels that were observed after an OGTT that provided no AA. In the later catabolic phase of the postprandial follow-up, the insulin concentration decreased, and the metabolism switched to lipolysis and ketogenesis for energy supply, as reflected by the delayed increases of NEFA, glycerol, FFA and ketogenesis-related products (3-hydroxybutanoic acid, acetoacetate and 2-hydroxybutanoic acid). In the same study, participants also underwent an OGTT, but differences with a mixed meal were only explored for glucose and insulin with the OGTT eliciting a higher but shorter insulin peak. 

Pellis et al. conducted a 5-week nutritional intervention (mix of anti-inflammatory compounds) followed by an HF challenge meal combined with metabolomics, targeted proteomics and classical clinical biomarker analysis in healthy overweight men with mildly elevated C-reactive protein levels (*n* = 36) [72]. The postprandial challenge enhanced the capacity of detecting a significant response to the intervention, as more than 50% of the affected features were detected exclusively during the test and not at the basal (fasting) state. Moreover, when comparing fasting and fed states, 106 metabolites were significantly changed. Within their 6-h follow up, they reported metabolic shifts similar to those of Wopereis et al. [63]. AA and glycolysis products increased during the first hours of the follow-up before decreasing to their baseline levels or slightly below. On the other hand, metabolites related to lipolysis (glycerol or long-chain FA) and ketogenesis increased after a lag time. Overall, six different clusters were identified among metabolites with distinct postprandial kinetics. 

Most studies had a short postprandial follow-up, and thus not all could report late postprandial changes; however, their results were consistent with those reported above. The study by Shrestha et al. explored the postprandial response to a complex meal (refined bread, cucumber and a non-caloric orange drink) in 19 clinically healthy postmenopausal women with a 3-h follow-up [66]. Following meal intake, the authors found that the metabolomics response was in line with the expected fasting-to-fed transition, including a shift in metabolic pathways from catabolic (fatty acid oxidation) to anabolic (suppression of ketogenesis and lipolysis) conditions with decreased acylcarnitine and ketone bodies levels and an increase followed by a return to baseline for pyruvate, lactate and AA. In the study by Mathew et al., 202 metabolites were assessed using a targeted metabolomics approach on male individuals (*n* = 11) [68]. Among them, 48 were differentially affected by a complex challenge meal conducted during the Ramadan period, after an interval of fasting lasting from sunset to sunrise. Although the population was heterogeneous (healthy, but with highly variable BMI) and the test meals were not fully standardized, several metabolites showed consistent changes 2 h after meal ingestion compatible with the expected postprandial changes. Thus, several AA increased (asparagine, arginine, alanine, glutamate, proline and phenylalanine), which was also the case for methionine sulfoxide, an oxidative product of methionine suggested to be a biomarker for oxidative stress in a challenge situation like the present one. Bile acids were also reported to be upregulated. The shift from fatty acid to glucose oxidation was indicated by decreased long-chain acyl-carnitine levels, concomitant with an increase in glucose and a drop in FFA blood levels. Yu et al. performed a metabolomics analysis on 123 healthy individuals in fasting state and 2 h following a balanced macronutrient challenge meal and reported on the great diversity of metabolic pathways affected [55]. A total of 1130 features were significantly changed between the fasted and fed state in both the HILIC and C18 LC-MS analyses. Pathway analysis identified several key routes associated with the postprandial response consistent with previous results, including several FA related pathways (with a significant decrease of linoleic acid) and bile acid synthesis (with decreased taurine and cholic acid). Moriya et al. reported that the ingestion of a traditional Japanese meal in healthy adults (*n* = 10) induced, at 1-h after meal intake, a shift from lipolysis to glycolysis with reduced levels of glycerol, long-chain fatty acids and increased glycolysis products, such as pyruvate [67]. Increased bile acids and AA levels were also increased following their absorption. 

In addition to these expected postprandial changes, more diverse metabolic shifts were also reported. TCA cycle intermediates, such as citrate, malate, succinate and α-ketoglutarate were frequently reported to be modified after challenge meals [55,63,66,67,72]. The direction of these changes was variable depending on the study and the metabolite considered with, however, an overall increasing trend. Regarding the specific lipid response to challenge meals, Morris et al. performed lipidomics profiling of 40 healthy individuals during a fitness test and 5-h after an HF challenge meal, provided in milkshake form [64]. Their results highlighted lysophosphoethanolamines, phosphoethanolamines, phosphoglycerides and ceramides among the metabolites with postprandial profiles most affected by the lipid challenge. More interestingly, among these metabolites, five lipid species (lysophosphatidylethanolamine (LPE) C18:2, LPE C18:1, phosphatidylethanolamine (PE) C36:2, PE C36:3 and one ceramide species) were actually predictive of fasting and peak postprandial TG values, which are known to be independent risk factors for cardiovascular diseases [77]. In addition, their results also supported the idea that post-meal profiles are more informative than fasting profiles, since the HF challenge revealed more metabolites significantly affected by fitness level (up to 52 metabolites at 180 min) compared in the fasting state (only nine metabolites).

### 5.2. Main Metabolic Shifts Induced by Mixed Meals in Individuals with Increased Cardiometabolic Risk

The metabolomics response to a mixed challenge meal has also been used to explore the subtle metabolic differences between populations with different cardiometabolic risks (Table 3). The postprandial follow-up is of particular interest in this case as it highlights additional differences compared to the fasted state, as discussed previously, and also because the postprandial response is considered to favor the initiation of atherosclerosis and diabetes in people with cardiometabolic risk.

Overall, most studies reported a decrease in phenotypic flexibility after the meal challenge in populations with increased cardiometabolic risk [56,57,58,59,62,63,66,69,70]. A decrease in flexibility was characterized by an altered response of the lipid and energy postprandial metabolism consisting of a blunted suppression of lipolysis and ketogenesis in the early hours following food intake and later on, in the post-absorptive state, of a blunted shift back to lipid oxidation and ketogenesis for energy fueling (Figure 1). 

Using dynamic metabolomics changes following a challenge meal, clinically healthy individuals were classified into subgroups with different cardiometabolic risk levels associated with contrasted postprandial responses. Fiamoncini et al. applied plasma metabolomics following the PhenFlex drink to determine metabotype clusters among 70 healthy individuals before and after a 12-week caloric restriction (20%) intervention [62]. Using multivariate statistical analyses, individuals were classified into two different metabotypes (A or B). Interestingly, individuals could not be classified in the fasting state, again highlighting the interest of the postprandial paradigm. The key metabolites involved in cluster definition were related to lipolysis (FFA and glycerol), β-oxidation (acylcarnitines) and ketogenesis (3-hydroxyutyrate). All those metabolites decreased slightly during the first 2 h following meal ingestion and then increased continuously before reaching a peak between 6 and 8 h. However, the magnitude of these postprandial changes was different between metabotypes with a blunted increase in metabotype B compared to metabotype A. Based on glucose clearance, BCAA and other metabolite levels, NMR urine analysis and dietary intake data, the authors considered individuals from metabotype B to be prediabetic-like, with modestly impaired insulin action (and higher visceral fat) despite the fact that they were initially classified as healthy according to the standard criteria. Regarding the caloric restriction intervention, no significant differences in postprandial responses were observed when considering the overall study population. However, when stratifying according to metabotypes, metabotype B showed a significantly improved glycemic response following the intervention. This suggests that deeper metabolomics-based phenotyping may help to identify metabotypes with different responses to nutritional interventions, even if these effects remain hidden to the mean. 

Shrestha et al. explored the postprandial response to a complex meal in healthy postmenopausal women as described previously [66]. Although the participants had a normal glucose tolerance, the authors found that two groups of participants could be identified on the basis of their insulin peak after the meal [78]. Several metabolic features suggested that individuals from the postprandial hyperinsulinemic group could be prone to early IR, like a blunted decline in several acylcarnitines, including C3 and C5 acylcarnitines, which are BCAA catabolic byproducts. Similarly, in the same group, a reduced decline in some phosphatidylcholines (PCs) was interpreted as a signal of major perturbation in the lipoprotein metabolism during the post-absorptive period. 

Other studies specifically investigated the differences between healthy and T2D subjects. Wopereis et al. measured postprandial response to the PhenFlex drink in healthy men (*n* = 20), as discussed in the previous section, as well as in T2D subjects (*n* = 20) [63]. Their results confirmed that differences between groups were exacerbated in the post-challenge condition with 58 biomarkers significantly modified between healthy and T2D groups compared to the fasted state (18 biomarkers modified). A blunted profile in FFA and glycerol, suggesting a lower suppression of the lipolysis rate, was reported in T2D subjects. Similarly, 3-hydroxybutanoic acid and acetoacetate, both involved in ketogenesis, exhibited fewer changes in T2D compared to healthy subjects. 

In a larger scale study, Li-Gao et al. compared the targeted metabolomics profile of normal glucose tolerant (NGT; *n* = 176), impaired fasting glucose (IFG; *n* = 186) and T2D (*n* = 171) individuals in response to a balanced liquid mixed meal [58]. At 150 min after the meal challenge, a profile of four metabolites assessed at the postprandial state was able to distinguish the T2D group from the NGT group with a similar efficiency to a fasting profile based on 12 metabolites. Thus, by submitting the individuals to the controlled meal challenge, the separation between T2D and NGT individuals was greatly enhanced, lending credence to the clinical usefulness of non-fasting metabolites as discriminant biomarkers of metabolic health and disease. The four-metabolite profile included glycine, lysophosphatidylcholines (LPC) a C17:0, acylcarnitines C16:1 and C4:1 (the latter two were ~20% higher in the T2D than the NGT group at 150 min, which is consistent with the hypothesis of a blunted decrease in β-oxidation following food intake). The authors also built another metabolite profile to distinguish T2D from NGT groups based on the response ratio between the fasting and postprandial levels, which included 16 features. Most of these metabolites were acylcarnitines and, more specifically, short-chain acylcarnitines, some of which (acetylcarnitine and C2) were already known to be associated with T2D. Despite these encouraging results, the precise meal composition was not presented, and thus conclusions about the metabolites discussed above should be interpreted with caution. 

Kumar et al. conducted a plasma untargeted metabolomics analysis on healthy controls, first degree relatives of T2D patients, overweight and prediabetes subjects following a complex challenge meal [69]. They reported altered levels of lysophospholipids and monoacylglycerol in the overweight and prediabetes groups with overall lower fasting and post-absorptive levels. 

In addition to metabolic dysregulations specifically associated with T2D, Yu et al. compared healthy controls (*n* = 123) with subjects with a range of cardiometabolic abnormality (*n* = 226), including obesity, hypertension, T2D and metabolic syndrome, 2 h after the ingestion of a mixed liquid meal [59]. The meal challenge resulted in changes in 1756 features in the cardiometabolic group vs. 1383 in the control group. The authors interpreted this difference as a result of a lower metabolic flexibility in the disease group. Postprandial changes were different in the cardiometabolic group as compared to the control group for 22 metabolites, including acylcarnitines and phospholipids, pointing to the importance of the postprandial lipid metabolism in cardiometabolic diseases. 

Alteration of the lipid metabolism was also reported in populations without overt disease but with increased cardiometabolic risk. Adamska-Patruno et al. used a high and a normal carbohydrate liquid meal test with a 3-h follow-up to discriminate healthy men with a genetic low vs. high predisposition to T2D [56]. Interestingly, the high carbohydrate liquid meal was devoid of fat; however, it did contain protein, therefore, eliciting more complex effects than a simple OGTT. The experiment conducted did not show significant differences in the fasting metabolite concentrations between genotypes. In contrast, the postprandial metabolome following the meal challenge tests uncovered several metabolic differences. Following the high carbohydrate meal, the high-risk group showed a blunted metabolic postprandial response when compared to the low risk individuals, with lower AUCs for most metabolites. In particular, postprandial changes in lipid metabolism differed between groups with respect to phospholipids, lysophospholipids, sphingolipids, arachidonic and oleic acids and their metabolites. In comparison, the postprandial metabolite profile after the normal carbohydrate meal intake, with a higher fat and protein content, elicited a metabolic response that appeared less unhealthy in the high risk group compared to the low risk group. Interestingly, irrespectively of the meal consumed, the postprandial acylcarnitine AUC in the high-risk group was reduced, suggesting a compensatory increase in fatty acid oxidation that could prevent lipid accumulation, as already seen in nondiabetic TCF7L2 HR-genotype carriers (TCF7L2, responsible for the T2D predisposition) [79]. From this point of view, the lower postprandial acylcarnitines appears to be a positive feature, since the increased tissue accumulation of acylcarnitine has been implicated in the activation of proinflammatory pathways involved in IR and T2D development [80,81]. Thus, the authors hypothesize that this compensatory mechanism could be responsible for a delayed drift towards overt T2D, at least in part in these glucose-tolerant and insulin-sensitive subjects.

The metabolomics postprandial follow-up of mixed meals highlighted differences according to cardiometabolic risk status not only in the lipid and energy metabolism but also in other metabolic pathways, such as those related to AAs and bile acids. Postprandial AA kinetics have been reported in several studies to be significantly different between groups with contrasted cardiometabolic risk with an overall trend for an enhanced postprandial increase associated with poorer cardiometabolic status [57,63,66,71], despite some inconsistencies between studies [57,70]. 

Fazelzadeh et al. compared the plasma metabolome after a complex meal in lean and obese participants, before and after a weight-loss intervention [71]. They reported distinct kinetics between lean and obese groups for several AA and 2-hydroxyisovalerate (a BCAA degradation product) with the latter showing an enhanced postprandial peak in the obese group. This finding could be the result of an impaired action of the BCKDH complex, responsible for the oxidation of this metabolite that has been associated with IR [52]. In addition, enhanced AA postprandial increases were associated with T2D compared to healthy participants [63], while a reduced AA decline following the initial surge was reported in women with a worse insulin profile compared with their counterparts [66]. Contrasted preliminary findings were also obtained by Bastarrachea et al. in a study aiming at explaining the inter-individual variations in risk of cardiovascular diseases, T2D and other cardiometabolic diseases on the basis of the variation in flexibility and efficiency in disposing of a regular liquid meal bolus [57]. The authors reported postprandial metabolomics results on a pilot study, including 16 healthy females with either low BMI (*n* = 8, BMI = 24) or high BMI (*n* = 8, BMI = 32). Among the different metabolites that discriminate between low and high BMI, the women with higher BMI had blunted postprandial increases in the vast majority of AA (alanine, arginine, asparagine, glutamine, glycine, histidine, isoleucine, leucine, lysine, methionine, proline, serine, threonine, tryptophan and valine) but higher postprandial increases in certain others (aspartic acid, cysteine, glutamic acid and phenylalanine)—this second finding being more in line with other studies. 

Regarding the bile acid metabolism, Bondia-Pons et al. reported interesting findings in a study exploring the environmental vs. genetic determinants of obesity in 16 healthy monozygotic twin pairs discordant for weight that consumed a standardized McDonald’s Big Mac Meal^TM^ [70]. This study design was close to that used by Rämö et al., which used the OGTT instead of a complex challenge meal [37] and further illustrated the potential of twin studies to explore the contribution of genetics vs. environment in the postprandial response. Despite the potential of the postprandial metabolome exploration to reveal metabolic differences, the authors found that within-pair similarity was the dominant factor in the metabolic postprandial response independently of the acquired obesity. They found modified bile acid profiles, two of which, glycine cholic acid (GCA) and glycine lithocholic acid (GLCA), had a higher concentration at 120 min after meal intake in heavier compared to lighter co-twins, suggesting that the response to the nutritional challenge was primarily driven by the acquired obesity independently of genetic factors. Furthermore, they provided evidence of a specific association between the postprandial changes in glycine ursodeoxycholic acid (GUDCA) and liver fat content as well as insulin sensitivity, although the precise mechanism could not be elucidated in this particular study. More recent evidence supports the role of this particular bile acid in the IR control, given that metformin has been shown to modify gut microbiota composition and increased GUDCA, which antagonize the intestinal farnesoid X receptor to improve hyperglycemia in diabetic patients [82].

## 6. Statistical Considerations: Which Statistical Tools for Metabolomics Postprandial Kinetics Analyses?

Metabolomics-based challenge test data were obtained from various experimental designs (crossed or nested, multilevel (before/after challenge) or longitudinal, parallel or cross over and balanced or unbalanced), as presented by Ulaszewska et al. [18]. Since challenge tests aim at describing dynamic changes, the information consists in performing repeated measurements on volunteers and collecting samples at different time points. Furthermore, to obtain a broad coverage of the metabolites included in biological samples, untargeted analytical methods are used to generate metabolomics data, which results in high-dimensional data. 

**Figure 2:**
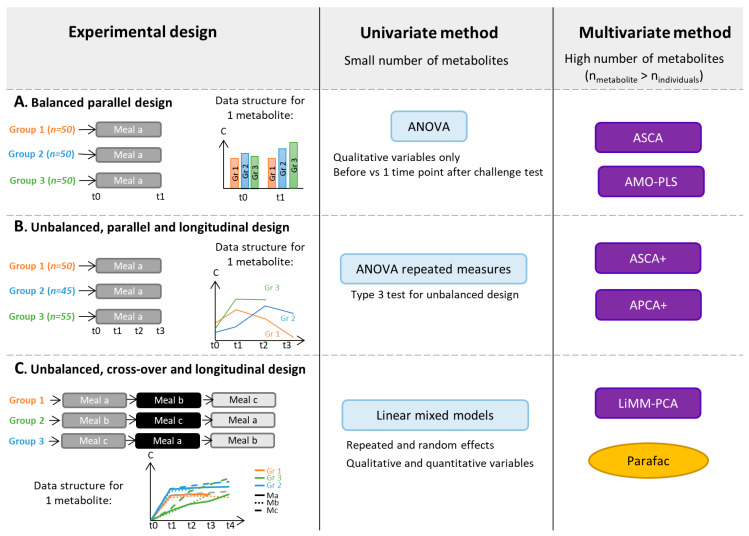
Selection of statistical methods that can be applied to metabolomics data obtained from challenge test studies with different experimental designs: Balanced parallel designs (**A**), unbalanced, parallel and longitudinal designs (**B**), unbalanced, cross-over and longitudinal designs (**C**). AMO-PLS: ANOVA-Multiblock Orthogonal Partial Least Squares, APCA: ANOVA-PCA, ASCA: ANOVA-Simultaneous Component Analysis, C: Metabolite concentration, LiMM-PCA: Linear Mixed Models-PCA, n_individuals_: number of individuals included in the experiment, n_metabolite_: number of metabolites quantified in the experiment, PARAFAC: Parallel Factor Analysis and t: Time.

Statistical analysis of metabolomics-based challenge test data raises several methodological challenges, i.e., the number of features is much larger than the number of volunteers, measures on the same volunteer are not independent, and spectral features are colinear. Therefore, it might be difficult to select the most appropriate statistical methods accounting for both study design and high-dimensional collinear features. With progress made in the field of omics, promising statistical methods are becoming available for more advanced designs. In the last few years, new methods have filled the gap in analyzing challenge test-based data for classification and discrimination purposes. We refer the reader to an existing review dedicated to statistical considerations associated with challenge tests and metabolomics for an exhaustive list of these methods [15], while we will focus on more recent advances, especially in the multivariate analysis field, and on the practical use of these methods (Figure 2). 

Univariate analyses can be used to separately analyze each metabolite and therefore tackle problems due to the large number of features and collinearities between spectral features. Paired *t*-tests [83] can be used to assess differences between the means of two matched groups of volunteers, e.g., data from before/after a nutritional challenge, as illustrated in Figure 2A. For instance, in the study of Liu et al. [46], control and hyperlipidemic patients underwent an OGTT. Through a targeted GC-MS analysis, 26 plasma metabolites were quantified in blood samples collected before and 2-h after OGTT. The authors performed paired *t*-tests to compare the average concentration of each metabolite before and after OGTT. The repeated measurement ANOVA [84] can be applied in a parallel longitudinal study (see Figure 2B).In a study conducted by Kumar et al. [69], four groups of participants, classified based on their health status and T2D history, underwent a mixed meal challenge with blood samples collected from 0 to 120 min, every 30 min (study design similar to Figure 2B). An untargeted metabolomics analysis was performed based on LC-MS. Repeated measures ANOVA were performed to explore the effect of time and risk factor on metabolite levels by selecting age and sex as covariates. Repeated measurement ANOVA uses the same conceptual framework as the classical ANOVA [85] but includes individuals as random effects.Paired *t*-test and repeated ANOVA relies on assumptions of normality of the residual distribution and homoscedasticity of variances and covariances, which can be difficult to assume or to test within high dimensional settings. Moreover, ANOVA estimators are biased in the case of an unbalanced design, e.g., with missing data (see Figure 2B,C for examples), and ANOVA makes the additional assumption that the matrix between observations on the same individual is of spherical covariance. 

Linear mixed models are more general than ANOVA and relax the assumption of sphericity. They can be used for advanced unbalanced multifactorial designs. For instance, explanatory variables can be qualitative as well as quantitative. It can be more accurate to treat time as a continuous variable, particularly when several time points are used as in Müllner et al. [40]. In this study, four groups of adolescents with different BMI and IR statuses underwent an OGTT. Blood samples were collected before and at seven time points after the challenge test.Metabolites were analyzed by NMR. A linear mixed model was fitted for each of the 49 identified and quantified metabolites to assess the effects of time and group with several adjustment factors. In univariate settings, to account for the multiple comparison problem, corrections of the *p*-value can be applied to control the false positive findings (Type I error). Widely used corrections include the Bonferroni [86] or False Discovery Rate (FDR) methods [87]. A general introduction of repeated measurement ANOVA and linear mixed models is given in Govaerts et al. [88].

To take full advantage of measurements in whole sets of metabolites, the application of multivariate methods can be beneficial. However, commonly used methods for metabolomic data analysis (unsupervised Principal Component Analysis (PCA), and supervised Projections to Latent Structures with Discriminant Analysis (PLS-DA)) are not suitable for multifactorial designs, such as the repeated measures generated in longitudinal challenge test studies.Supervised methods use classes (‘labels’) of volunteers (for instance, Control/Case or Unsupplemented/Supplemented diet) to adjust models whereas unsupervised methods do not. Therefore, several approaches based on a combination of ANOVA and multivariate methods have been developed to analyze data from complex designs (see Guisset et al. 2019 for a review [89]). In the ANOVA step, the data matrix is broken down into effect matrices that correspond to each term of the ANOVA model (the main factors of the study design or interactions). The multivariate method is then applied to reduce data dimensionality.For instance, A-SCA (ANOVA-Simultaneous Component Analysis [90]) is used to perform an PCA on each pure effect matrix, ANOVA-PLS [91] is based on a PLS analysis of each augmented effect matrix, whereas the AMOPLS (ANOVA Multiblock Orthogonal PLS [92])) allows the simultaneous analysis of all the augmented effect matrices. Augmented effect matrix corresponds to the sum of pure effect matrix and residual matrix. These methods can be applied to designs similar to Figure 2A. For instance, Rådjursöga et al. [21] applied the ANOVA-PLS method to study NMR-based metabolomics data from blood samples of 24 volunteers, who ate alternatively two types of breakfast on four occasions. For the same reason as above, ASCA+ and APCA+ methods [93] have been developed, in particular, for experimental unbalanced designs with fixed categorical factors (see Figure 2B). In those two methods, the general linear model replaces ANOVA in the first step.Martin et al. further generalized ASCA+ to linear mixed models in the LiMM-PCA (Linear Mixed Models-PCA) method, enabling the inclusion of both fixed and random effects [94]. LiMM-PCA is well adapted to analyze data from advanced cross-over challenge tests, such as that presented in Bütikofer et al., in which untargeted LC-MS was applied to serum samples collected at five time points after meal intake (three cross-over strategies) from normal weight and obese men [74] (see Figure 2C for an example of such study design).

The domain of multi-way analysis ([95]) is also an appropriate framework for the analysis of data matrixes from longitudinal designs (see Box 1 in [18] or Figure 2C). Two-dimensional data matrixes can be folded into a cube, where the dimensions of the cube are the individuals, the metabolites and the sampling time points. Several methods exist to analyze multi-way data, e.g., the PARAFAC (Parallel Factor Analysis) approach [96].

As summarized in Figure 2, the analysis of multivariate data from nutritional challenge tests can be based on ASCA or AMOPLS in the case of balanced and parallel designs and on ASCA+ and APCA+ in the case of unbalanced and parallel designs. In the case of more complex designs, such as cross over and unbalanced data, LiMM-PCA should be favored. Indeed, mixed linear models offer a general framework to analyze data from multifactorial experimental designs involving both fixed and random categorical and continuous factors of unbalanced data.

## 7. Conclusions and Perspectives

The studies reported and discussed in this review provide a body of relevant and definitive evidence that underscores the importance of the use of challenge meals combined with metabolomics phenotyping to characterize phenotypic flexibility, a core feature of metabolic health. This paradigm proved to be efficient for identifying differences between populations, groups and individuals (including clinically healthy individuals) that were not visible at the fasting state. 

Generally, the metabolic profiling studies after either OGTT or mixed meals highlighted consistent effects on key metabolic pathways controlled by insulin action: the fasted-to-fed early energetic shift from lipid oxidation and ketogenesis to glucose oxidation was visible through a decrease in glycerol, FFA, acylcarnitines and ketone bodies as glycolysis products increased.In the late postprandial period, the metabolism shifts back to lipid oxidation, and the related metabolites bounce back. Following OGTT, insulin action inhibits proteolysis as indicated by decreased AA levels, whereas most AA exhibit typical absorption curves after the ingestion of AA-containing mixed meals. When comparing healthy vs. individuals with expected reduced phenotypic flexibility (often associated with increased cardiometabolic risk), a blunted response on most key postprandial pathways was reported. 

Many challenges remain to be addressed. First, the specificities of the dataset generated by metabolomics combined with a kinetic follow-up require specific statistical tools that call for further methodological development. Second, there is considerable heterogeneity between challenge meal composition and food matrix complexity that makes achieving optimal study reproducibility and between-study comparisons difficult. Finally, each challenge test category has its own advantages and drawbacks, ranging from high standardization but poor ability to mimic full postprandial response for the OGTT to a significant capacity to elicit a response close to real life settings but very high compositional variability for complex challenge meals.

However, if these challenges are correctly addressed, the dynamic postprandial follow-up of the metabolome holds great promise and could contribute more widely to nutritional science. In the field of precision nutrition, the postprandial period has been used to develop personalized nutrition guidelines based on individual response profiles [97], without using metabolomics to assess the food intake response up to now.The addition of this omics approach could provide further insights into the postprandial response, thereby improving metabotype phenotyping and furthering guidelines fitting individual or group specificities. Other novel analytical methods could also be combined with metabolomics to provide a better understanding of the determinants of the food intake response and characterize inter-individual variability. For instance, very recently, targeted plasma metabolomics were combined with genetic analyses to explore the genetic determinants of the meal response [98].This quite unique study design calls for repetition and would gain from wider metabolite coverage. Challenge meal studies could also benefit from recent advances in the field of multi-omics data integration [99,100,101]. Some of the studies discussed in this review included transcriptomics [57,71] or proteomics [72] in addition to metabolomics. However, they did not perform any deep multi-omics data integration, which could provide a better understanding of the postprandial response, in particular, regarding the mechanistic aspects.

## Figures and Tables

**Table 1:** Summary of reviewed studies investigating, with a metabolomics approach, the response to OGTT in humans.

**OGTT Response in Healthy Individuals**					
**Subjects**	**Samples after OGTT**	**Analytical Platform**	**Response to the OGTT vs. Fasting**		**Refs.**
Men and women (*n* = 16)	Plasma at 0, 30, 60, 90 and 120 min	UPLC-qTOF-MS	↓ saturated and monounsaturated FFA ↓ C10:0, C12:0 and C14:1 acylcarnitines ↑ PUFA ↑ bile acids ↑ lysophosphatidylcholines.		[31]
Men and women (*n* = 6)	Plasma at 0, 15, 30, 45, 60, 75, 90, 105 and 120 min	GC-MS	↓ fatty acids ↓ AA and related metabolites (isoleucine, valine, tyrosine, phenylalanine, methionine, threonine, lysine, arginine, glycine, ornithine, 4-hydroxyproline) and threonic acid ↑ alpha-tocopherol, cystine		[33]
**OGTT Response Depending on IR Status**					
**Subjects**	**Samples**	**Analytical Platform**	**Response to the OGTT vs. Fasting**	**Comparison vs. Healthy Individuals**	**Refs.**
Healthy men and women (*n* = 22): Young adults from Metabolic Abnormalities in College Student study, aged 18–30 year old Validation cohort (*n* = 25): Participants from Framingham Offspring study, aged 40–49 year-old Subjects with impaired glucose tolerance (*n* = 25): Participants from Framingham Offspring study, aged 40–50 year old, 2 h glucose concentration between 140 and 199 mg/100 mL	Plasma at 0, 30, 60, 90 and 120 min	LC-MS	18 metabolites changed during the OGTT: ↓ AA and related metabolites (valine, leucine/isoleucine, phenylalanine, tyrosine, histidine, lysine, arginine, methionine, ornithine and citrulline) ↓ urea cycle intermediates: ornithine and citrulline ↓ glycerol ↓ ketogenesis product (β-hydroxybutyrate) ↓ hypoxanthine ↑ glycolysis products (pyruvate and lactate) ↑ bile acids ↑ hippuric acid	Higher fasting insulin vs. lower fasting insulin groups: Smaller ↑: lactate and bile acid Smaller ↓: AA (leucine/isoleucine, valine and methionine), β-hydroxybutyrate and glycerol	[32]
Nondiabetic individuals (*n* = 377): Framingham Offspring cohort participants, mean age 57 years, Mean BMI = 30 kg/m^2^ IR was defined as the top quartile of HOMA-IR from the entire cohort free of diabetes at the 5th examination cycle.	Plasma at 0 and 120 min	LC-MS	91 metabolites significantly changed with OGTT: ↓ AA (including BCAA and AAA) ↓ β-hydroxybutyrate ↓ TCA cycle intermediates, ↓ unconjugated bile acids ↓ urea cycle metabolites (citrulline, ornithine and arginine) ↓ nucleic acids derivatives (hypoxanthine, xanthine etc.) ↓ serotonin derivatives and B vitamins ↑ glycolysis products (lactate, phosphoenolpyruvic acid and phosphoglycerate) ↑ conjugated bile acids	IR vs. IS groups: Smaller ↓ for β-hydroxybutyrate, isoleucine and pyridoxate Smaller ↑ for lactate Higher ↓ for orotate Changes in taurodeoxycholates, niacinamide and ornithine associated with HOMA-IR with a blunted response for individuals with greater IR	[34]
Non-diabetic, IR individuals (*n* = 470): Men, 70.6 years old	Plasma at 0, 30 and 120 min	UPLC-TOF-MS	Not applicable	35 metabolites associated with IR: 7 glycerophospholipids, 6 glycerolipids, 4 glycerophosphoethanolamines, 6 unsaturated FAs, 4 acylcarnitines, 2 bile acids and one each of monosaccharide, peptide, SFA, steroids, imidazopyrimidine and propranolol IR vs. IS individuals: Smaller ↓: oleate, palmitoleate, C10-carnitine and C12-carnitine Lack of ↓: LPE 18:1, LPE 18:2 and LPE 20:4 ↑ Deoxycholate-glycine ↑ for both IR and IS subjects followed by a smaller ↓ to baseline in IR subjects: hexoses	[39]
Healthy individuals (*n* = 4745): middle-aged Finnish Replication cohort (*n* = 595): senior Finnish participants IS-NGT group: fasting insulin at the bottom quartile of NGT and fasting glucose < 5.6 mmol/L and 2-h glucose < 7.8 mmol/L) IR-NGT group: fasting insulin at the top quartile of NGT and fasting glucose < 5.6 mmol/L and 2-h glucose < 7.8 mmol/L)	Serum at 0, 30, 60 and 120 min	NMR	↑ glycolysis intermediates (pyruvate and lactate) ↑ TCA intermediate (citrate) ↓ BCAA (isoleucine, leucine an and valine) and AAA (phenylalanine and tyrosine) ↓ ketone bodies (beta-hydroxybutyrate and acetoacetate). ↓ Acetate, TG and glycerol	IR-NGT vs. IS-NGT groups: Smaller ↑: glycolysis products (pyruvate, lactate and alanine) Smaller ↓ for ketone bodies (β-hydroxybutyrate and acetoacetate), BCAA, glycerol and VLDL and HDL TG Similar findings were reported in individuals with prediabetes and diabetes compared to the IR-NGT group.	[35]
Healthy men and women (*n* = 246): Non-obese (BMI < 30 kg/m^2^), young adults aged 18–35	Plasma at 0 and 120 min	LC-MS	405 lipids significantly perturbated following the OGTT. ↓ sphingolipids and SM ↓ acylcarnitines, especially C14:2 ↓ LPC, LPE and lysophosphatidylinositol ↓ DG and TG species containing MUFA or SFA ↑ lysoetherphospholipid species	Association with HOMA-IR: Positive association: 12 ceramides, 10 deoxyceramides, 22 PE, 6 PS, 19 DG and 42 TG. Negative association: 3 alkylphosphatidylcholine, 16 alkenylphosphatidylcholine, 6 medium- to long-chain acylcarnitine, 9 cholesteryl ester containing omega-3 and omega-6 polyunsaturated fatty acids and 9 hexosylceramide species. Cholesteryl ester (20:4) displayed the strongest association with 17.8% decrease per HOMA-IR unit Association with Insulin AUC: 5 Medium chain acylcarnitines, 25 TG, 10 DG, 15 LPC, 10 PE and 4 PS	[36]
NGT group (*n* = 234): fasting glucose < 5.6 mmol/L and OGTT 2 h glucose < 7.8 mmol/L Prediabetes group (*n* = 281): 5.6 ≤ fasting glucose < 7.0 mmol/L or 7.8 ≤ OGTT 2 h glucose < 11.1 mmol/L Newly diagnosed T2D group (*n* = 66): fasting glucose ≥ 7.0 mmol/L or OGTT 2 h glucose ≥ 11.1 mmol/L	Plasma at 0 and 120 min	LC-MS	35 increased metabolites (NGT: 18, prediabetes: 23, T2D: 13): ↑ tauropine ↑ methane metabolism related metabolites ↑ glycolysis related metabolites (3-phospho-D-glycerate) 45 decreased metabolites (NGT: 36, prediabetes: 29, T2D: 18) ↓ AA-related metabolites (glutamate and citrulline) ↓ purine metabolism metabolites: xanthosine, AMP and hypoxanthine ↓ fatty acids oxidation metabolites	T2D vs. NGT groups: 22 metabolites significantly different ↑ AA: glutamate and homocysteine ↑ TCA cycle and glycolysis intermediates: succinate, malate, pyruvate Smaller ↓ pentose phosphate pathway (D-glycerate) Smaller ↓ galactose metabolism (alpha-D-galactosyl-(1-3)-1D-myo-inositol)	[38]
Adolescent aged 10–18 year-old Group 1: Lean, NGT, IS group (*n* = 21) Group 2: Obese, NGT, IS group (*n* = 18) Group 3: Obese, NGT, IR group (*n* = 20) Group 4: Obese, IGT, IR group (*n* = 23)	Plasma at 0, 15, 30, 60,90 and 120 min	NMR		IR groups (group 3–4) vs. IS groups (1–2): ↑ BCAA (valine, leucine, isoleucine), AAA (tyrosine, phenylalanine) and lysine, 2-oxoisocaproic acid ↓ serine, glycine, myo-inositol and dimethylsulfone In obese groups (2–3-4) compared to the lean group (1): ↑ glutamate, alanine, pyruvate and O-acetylcarnitine ↓ acetate	[40]
**OGTT Response Depending on Obesity and BMI Status**					
**Subjects**	**Samples after OGTT**	**Analytical Platform**	**Response to the OGTT vs. Fasting**	**Comparison vs. Healthy Individuals**	**Refs.**
Obese group (*n* = 14): BMI 43.6 kg/m^2^ Lean group (*n* = 6): BMI of 22.4 kg/m^2^	Serum at 0, 30 and 120 min	GC-MS	In the obese group: 52 metabolites affected by OGTT No change at 30 min and ↓ at 120 min: β-hydroxybutyrate, glycerol, hypoxanthine and fatty acids Heterogeneity in AA and fatty acids profiles with overall ↓at 120 min	Obese vs. lean group: 16 metabolites significantly different (out of 59) 30 min delayed ↓: palmitic acid, lauric acid, oleic acid, pentadecanoic acid and stearic acid. 30 min ↑ asparagine, glutamate, taurine, tyrosine, isoleucine and leucine. 30 min lack of ↑: pyrophosphate, threonic acid, phenylalanine, serine, glyceric acid and aspartate.	[41]
Young college students aged 18–23 years Lean group (*n* = 15): BMI > 18.5 and <23 kg/m^2^ Obese group (*n* = 15): BMI > 27.6 kg/m^2^	Serum at 0, 30, 60, 90 and 120 min	UPLC–TQ–MS GC-MS	In lean subjects: ↓ fatty acids (C18:3, C18:2, C16:1 and C16:0) ↓ amino acids (BCAA, phenyalanine, tryptophan, alanine, proline, glycine, methionine, serine, arginine, threonine, asparagine and lysine) ↓ biogenic amines (taurine, creatine and GABA) ↑ niacinamide, tyrosine, histidine and glycerophophorylcholine	In obese vs. lean group: Smaller ↓ for most AA and fatty acids BMI, waist circumference, body fat and fat mass were positively associated with arginine, histidine and GABA OGTT postprandial changes. OGTT postprandial changes in palmitic acid, BCAA, phenylalanine and lysine were positively associated with fasting insulin and HOMA-IR in the obese group.	[42]
Obese individuals (*n* = 14) compared at: - baseline (mean BMI = 43.7 kg/m^2^) - after a 3-month weight loss program (mean BMI = 36.2 kg/m^2^) - after a subsequent 5-month weight maintenance phase (mean BMI = 34.9 kg/m^2^)	Serum at 0, 30 and 120 min	GC-MS	Not applicable	After weight loss and weight-maintenance phases compared to baseline in obese individuals: Higher ↓: AAA (tyrosine and phenylalanine), BCAA (leucine, isoleucine), FFA and glycerol Suppressed ↑: glutamate and glutamine	[43]
Young healthy twins (*n* = 274): monozygotic twin pairs (*n* = 64) and dizygotic twin pairs (*n* = 73) with either concordant BMI or discordant BMI	Serum at 0, 30 60 and 120 min	NMR	Response in the whole population: ↓ AA (BCAA, tyrosine, phenylalanine, histidine, glycine and glutamine) ↓ ketone bodies (β-hydroxybutyrate, acetoacetate and acetate) ↓ glycerol, fatty acids, total TG and VLDL TG ↑ glycolysis products (lactate and pyruvate) ↑ very large HDL-TG and small HDL-TG	Individuals with higher BMI and liver fat content vs. cotwins: Smaller ↓ isoleucine, SFA and MUFA, TG, small VLDL and small LDL	[37]
Adolescents (8–17 year-old) clinically healthy Lean group (*n* = 55): BMI percentile < 85th for sex and age) Overweight or obese group (*n* = 228): BMI percentile ≥ 85th for sex and age	Plasma at 0 and 60 min	LC-MS	Response in the whole population: ↓ medium and long-chain acylcarnitines, FFA, lipids, such as SMs, PCs and DGs ↑ hippurate	Obese or overweight vs. lean groups: Smaller ↑: medium and long-chain acylcarnitines, FA oxidation intermediates and FAs	[44]
**OGTT Response Depending on Lipidemic Status**					
**Subjects**	**Samples after OGTT**	**Analytical Platform**	**Response to the OGTT vs. Fasting**	**Comparison vs. Healthy Individuals**	**Refs.**
Healthy group (*n* = 35) HLP group (*n* = 35): TG > 1.7 mmol/L Total cholesterol >5.7 mmol/L	Serum at 0 and 120 min	UPLC-TQ-MS	Healthy group: ↓ methionine, aminobutyric acid, niacinamide, 4-hydroxy-l-proline, valine, GABA, glutamic acid, asparagine, tyrosine and allantoin) ↑ serine, taurine, cysteine and creatine In the HLP group: ↑ leucine, isoleucine, serine, histidine, lysine, γ-aminobutyric acid, taurine, cysteine and creatine ↓ methionine, dimethylglycine, aminobutyric acid, niacinamide, allantoin and creatinine	HLP vs. healthy groups: Higher ↑: cysteine, taurine, lysine, histidine and leucine. ↑ instead of ↓: GABA, tyrosine, asparagine, isoleucine and valine ↓ instead of ↑: dimethylglycine and creatine Smaller ↓: methionine Within the HLP group, IR vs. non IR subjects: Higher ↑: GABA, tyrosine, taurine, isoleucine, leucine and valine. Correlation within HLP group with IR: Isoleucine and GABA postprandial OGTT changes positively correlated to HOMA-IR	[45]
Healthy group (*n* = 50) HLP group (*n* = 38): TG > 1.7 mmol/L Total cholesterol > 5.7 mmol/L	Serum at 0 and 120 min	GC-MS	Healthy group: ↓ glycolysis (lactic acid) ↓ TCA cycle intermediates (citric acid and malic acid) ↓ ketogenesis product (β-hydroxybutyrate) ↓ pyroglutamic acid, α-hydroxybutyrate, pimelic acid and suberic acid ↑ cis-aconitic acid	Hyperlipidemic vs. healthy groups at 120 min: Smaller ↑: glycolysis products (pyruvate, phosphoenol pyruvate), TCA cycle intermediates (oxalic acid, isocitric acid, fumaric acid) and orotic acid ↓ instead of ↑: glycolysis product (lactate), TCA cycle intermediates (malonic acid, citric acid), sebacic acid, suberic acid, pyroglutamic acid, glycolic acid, α-hydroxybutyrate and caprylic acid ↑ instead of ↓: 2-hydroxyisocaproic acid	[46]

AMP: Adenosine monophosphate, AA: Amino acids, AAA: Aromatic amino acids, AUC: Area under the curve, BMI: Body mass index, BCAA: Branched-chain amino acids, DG: Diacylglycerol, FA: Fatty acid, FFA: Free fatty acids, GABA: γ-aminobutyric acid, HDL: High density lipoprotein, HOMA-IR: Homeostasis model assessment-Insulin resistance, HLP: Hyperlipidemic, IGT: Impaired glucose tolerance, IR: Insulin resistant, IS: Insulin sensitive, LDL: Low density lipoprotein, LPC: lysophosphatidylcholine, LPE: Lysophosphatidylethanolamine, MUFA: Monounsaturated fatty acids, OGTT: Oral glucose tolerance test, NGT: Normal glucose tolerant, PC: Phosphatidylcholine, PE: phosphatidylethanolamine, PS: phosphatidylserine, PUFA: Polyunsaturated fatty acids, SFA: Saturated fatty acids, SM: sphingomyelin, TCA: Tricarboxylic acid, TG: Triglycerides, T2D: Type 2 diabetes and VLDL: Very low density lipoprotein.

**Table 2:** Summary of reviewed studies investigating the mixed meal response in healthy individuals with a metabolomics approach.

Healthy Population						
Subjects	Samples	Analytical Platform	Response to Challenge Meal (vs. Fasting)	Main Meal Ingredients	Meal Macronutrient Composition (kcal, %E)	Refs.
Healthy men and women (*n* = 123)	Plasma at 0 and 2 h after meal intake	LC-MS	1130 features significantly different Key pathways affected in C18 analyses: - Bile acid synthesis (↓ taurine and ↓ cholic acid) - TCA cycle (↑ malic acid and citric acid) - Fatty acid metabolism - Linoleic acid metabolism (↓ linoleic acid) - Biotin metabolism - Valine, leucine and isoleucine biosynthesis Key pathways affected in HILIC analyses: - Primary bile acid synthesis - 1-carbon pool by folate - Cyanoamino acid metabolism - Sphingolipid metabolism - Ascorbate aldarate metabolism - Fatty acid elongation in mitochondria Phenylalanine metabolism	Mixed liquid meal Incaparina (vegetable protein mixture): 12 g, skim milk (lactose-free): 170 mL, safflower oil: 25 g, sugar: 52 g	520 kcal Carbohydrate: 52%E Lipid: 42%E Protein: 6%E	[55]
Healthy men and women (*n* = 100) age range: 19–71 years-old Classification in 10 groups according to gender, age and body fat percentage Optimal phenotypic flexibility group: 20–29-year-old men and women with low to normal body fat (<20%) Reduced phenotypic flexibility group: 60–70-year-old men and women with normal to high body fat (>20%)	Plasma at 0, 0.5, 1, 2, 4, 6 and 8 h after meal intake	GC-MS	“reduced phenotypic flexibility” group vs. “optimal phenotypic flexibility group”: ↑ 4-methyl-2-oxovalericacid, 3-methyl-2-oxovaleric acid, tyrosine, isoleucine	High fat liquid meal 400 mL beverage Palm olein: 12.4% (weight/weight), dextrose: 17.25%, protifar (Nutricia): 4.13%, vanilla flavor: 0.10%, trisodium citrate: 0.12%, sodium hydroxide: 0.08%, water: 66.12%	950 kcal Carbohydrate: 33%E Lipid: 59%E Protein: 8%E	[61]
Healthy men and postmenopausal women (*n* = 72): Mean age: 59.2 ± 4.2 y; Mean BMI: 29.7 ± 2.7 kg/m^2^	Plasma at 0, 1, 2, 4, 6 and 8 h after meal intake	LC-MS/MS	Key metabolites for the identification of two distinct metabotypes: Lipolysis: glycerol, FFA Ketegogenesis: 3-OH-butyric acid, β-oxidation: acetylcarnitine (C2), hexanoylcarnitine (C6), octanoylcarnitine (C8), decanoylcarnitine (C10), dodecanoylcarnitine (C12), miristoylcarnitine (C14) and the ratio of medium-to-long chain acylcarnitines In metabotype B (considered as prediabetic with decreased insulin sensitivity and higher visceral fat) vs. metabotype A: smaller ↑ after an initial ↓: lipolysis products (FFA), ketogenesis products (3-OH-butyric acid) and β-oxidation products (acylcarnitines)	High fat liquid meal 400 mL beverage Palm olein: 12.4% (weight/weight), dextrose: 17.25%, protifar (Nutricia): 4.13%, vanilla flavor: 0.10%, trisodium citrate: 0.12%, sodium hydroxide: 0.08%, water: 66.12%	950 kcal Carbohydrate: 33%E Lipid: 59%E Protein: 8%E	[62]
Healthy men and women (*n* = 40): aged 18–60 years	Plasma at 0, 1, 2, 3, 4 and 5 h after meal intake	LC-MS/MS-ESI	Metabolite with a fold change > 1.5 between 0 min and following time-point: At 60 min: N-C10:0(OH)-Cer(2H) and N-C26:0-Cer(2H) At 120 min: LPE a C18:2, LPE a C18:1, PE aa C36:2, PE aa C36:3 and N-C16:1-Cer, PG aa C36:2 At 180 min: PE aa C36:1, PG aa C34:1 and N-C24:0(OH)-Cer(2H), PG aa C36:2 At 300 min: N-C25:0(OH)-Cer LPE a C18:2, PE aa C36:2 and PE aa C36:3 were predictive of fasting and peak plasma TG concentrations following the challenge test.	High fat liquid meal Calogen (Nutricia): 100 mL, liquid Duocal (SHS Nutrition): 50 mL	533 kcal Carbohydrate: 8%E Lipid: 92%E Protein: 0%E	[64]
Healthy postmenopausal women (*n* = 19): Divided on 2 subgroups (A and B) based on insulin response. Subgroup B compared to subgroup A: Higher postprandial insulin response for similar glucose response	Serum at 0, 30, 45, 60, 90 and 180 min after meal intake	LC-MS NMR	73 metabolites significantly different ↓ acylcarnitines ↓ ketone bodies (3-hydroxybutyrate, acetone) ↑ and ↓ below baseline: leucine, isoleucine (and catabolic products), phenylalanine, methionine and threonine catabolic products ↑ and ↓ to baseline: alanine and proline, 2-hydroxyisovalerate (originates from ketogenesis and BCAA) ↑ and ↓: glycolysis products (pyruvate, lactate) ↑ and ↓: TCA cycle intermediates (succinate, citrate) Subgroup B vs. subgroup A at 180 min after meal intake: Higher ↑ just after meal intake: leucine ↑ followed by smaller ↓: AA (lysine, serine), creatinine Smaller ↓ acylcarnitines (C3, C4, C5, C16) No ↓ PCs (PC aa C28:1, PC ae C38:1, PC ae C40:1, PC ae C42:3)	Complex meal Refined wheat bread, cucumber: 40 g, noncaloric orange drink: 300 mL	For bread alone: 281 kcal Carbohydrate: 71.1%E Lipid: 16.6%E Protein: 12.8%E	[66]
Healthy men and women (*n* = 10) Aged 25–50 years old	Plasma at 1 h before meal and 1 h after meal intake	UPLC-MS/MS GC-MS	↑ glycolysis related products (pyruvate) ↑ primary and secondary bile acids (conjugated form) ↑ AA ↑ TCA cycle related products (malate, citrate) ↓ succinate ↓ lipolysis and β-oxidation related products (glycerol, fatty acids and 3-hydroxybutyrate) ↓ endocannabinoids	Complex meal Broiled salmon, pork cutlet, shao mai, Japanese omelet, kamaboko, ganmodoki, rice, tomato, lemon, vegetable mix, ginger, heavenly bamboo, carrot, taro, snow pea and bamboo shoot	763 kcal Carbohydrate: 53.2%E Lipid: 28.8%E Protein: 15.5%E	[67]
Healthy men (*n* = 11) Muslim volunteers of varying BMI and age performing Ramadan fasting	plasma at 0 and 2 h after meal intake	LC-MS/MS FIA-MS/MS	48 metabolites were significantly changed ↑ AA (asparagine, arginine, alanine, glutamate, proline and phenylalanine) ↑ methionine sulfoxide (degradation product of methionine with reactive oxygen species, potential marker of oxidative stress) ↑ glycine/taurine conjugated bile acid ↓ long-chain acyl-carnitine ↓ polyamine (spermidine and putrescine)	Complex meal Meal week 1: white rice: 100 g, egg pasta: 50 g, chicken meat: 150 g, bell pepper: 50 g, avocado: 50 g, whipping cream 30% fat: 20 g, orange juice: 200 mL, Italian salad: 100 g, rice pudding: 100 g, pita bread: 40 g, vegetable soup: 200 mL Meal week 4: white rice: 120 g, raisin: 15 g, hazelnuts: 15 g, yoghurt 3.5% fat: 150 mL, pita bread: 40 g, orange juice: 200 mL, lamb muscular meat with intermuscular fat: 200 g, lentil soup: 200 mL, white bread with grains: 13 g and Italian salad: 100 g	Meal week 1: 1097 kcal Carbohydrate: 38.5 E% Lipid: 38.2E% Protein: 23.3E% Meal week 4: 1322 kcal Carbohydrate: 37.8 E% Lipid: 42.3E% Protein: 19.9E%	[68]

AA: Amino acids, BMI: body mass index, BCAA: Branched-chain amino acids, Cer: ceramides, E: Energy, FFA: Free fatty acids, HILIC: Hydrophilic interaction liquid chromatography, LPE: Lysophosphatidylethanolamine, PC: Phosphatidylcholine, PE: phosphatidylethanolamine, PG: phosphoglycerides, TCA: tricarboxylic acid and TG: Triglycerides.

**Table 3:** Summary of reviewed studies investigating mixed meal response in populations with pre- or pathological conditions, with a metabolomics approach.

Subjects	Samples	Analytical Platform	Observed Response to Challenge Meal (vs. Fasting)	Comparison vs. Healthy Controls	Meal Ingredients	Meal Macronutrient Composition (kcal, %E)	Refs.
Healthy overweight men (*n* = 36): BMI: 25.6–34.7 kg/m^2^ Mildly elevated C-reactive protein (CRP) levels: 1.0–8.1 µg/L	Plasma at 0, 1, 2, 3, 4 and 6 h after meal intake	GC-MS	106 metabolites significantly modified Late ↑ lipolysis related products (long chain FFA and glycerol) Late ↑ ketogenesis related products (3-hydroxybutanoic acid and acetonacetate) Late ↑: succinate ↑ followed by ↓ below baseline: Glycolysis and TCA intermediates related products (pyruvate, citrate, α-keto-glutaric acid) ↑ followed by ↓ to baseline: most amino acids ↓ lactate ↓ uric acid	Effects of an anti-inflammatory dietary mix in a cross-over, double-blind intervention. 31 plasma features (metabolites and proteins) were significantly different between groups with 17 uniquely identified at the fed state compared to the fasting state.	Complex meal Dairy shake 500 mL: Custard: 300 mL, cream cheese: 150 mL and whipping cream: 50 mL	706 kcal Carbohydrate: 29.6 E% Lipid: 58.7E% Protein: 11.7E%	[72]
healthy women Low BMI group (*n* = 8): BMI = 25 High BMI group (*n* = 8): BMI = 32	Plasma at 0, 0.5, 3 and 5 h after meal intake	HPLC-ESI-MS		High BMI vs. low BMI group: Smaller ↑: alanine, arginine, asparagine, glutamine, glycine, histidine, isoleucine, leucine, lysine, methionine, proline, serine, threonine, tryptophan and valine Higher ↑: aspartic acid, cysteine, glutamic acid and phenylalanine ↓ acylcarnitine C14:3 (30 min) ↓ acylcarnitine (300 min)	Mixed liquid meal Ensure Plus^®^ (Abbott Nutrition, Lake Buff, Illinois, USA)	30% of the participant’s daily energy requirement based on total body weight and fat free mass (kg) Carbohydrate: 57%E Lipid: 28%E Protein: 15%E	[57]
Healthy men and women (*n* = 50) Monozygotic twins discordant for weight (*n* = 32): BMI difference >3 kg/m^2^ Monozygotic twins concordant for weight (*n* = 18): BMI difference < 3 kg/m^2^	Serum at 0, 0.5, 1 and 2 h after meal intake	GC-GC-TOFMS UPLC-QTOFMS UPLC-QqQMS		20 metabolites with a converging time profile (≠ at fasting but = at fed state): ↑ at fasting state in heavier co-twins: isoleucine and valine ↓ at fasting state in heavier co-twins: fatty acids 22 metabolites with diverging time profile (= at fasting but ≠ at fed state) ↑ at fed state in heavier co-twins: sugar derivatives (arabinitol) and organic acids (acetic acid) ↓ at fed state in heavier co-twins: lipids and bile acids	Complex meal McDonald’s Big Mac Meal^TM^ (Chicago, Illinois, USA) 1 Big Mac hamburger, French fries: 100 g, sucrose-sweetened Coca-Cola^TM^: 400 g	979 kcal Carbohydrate: 50 E% Lipid: 37 E% Protein: 13 E%	[70]
Lean group (*n* = 15): BMI: 23.0 Abdominally obese men group (*n* = 29): BMI = 30.3 This group underwent an 8-wk weight loss intervention or control intervention	Plasma at 0, 0.5, 1, 2, 3 and 4 h after meal intake	UPLC UPLC-MS/MC GC-MS ESI-MS NMR		Lean vs. obese: alanine, proline, threonine, histidine, methionine, 2-hydroxyisovalerate (degradation product of BCAA), phosphocholine and methylmalonic acid significantly different. Effect of the weight-loss intervention in the obese group: At the fed state, 11 metabolites were significantly changed before and after intervention: Oxylipins, glutamine, histidine, creatine, pyroglutamic acid, glucose and choline	Complex meal 2 muffins, 0% fat milk: 300 mL	1100 kcal Carbohydrate: 44%E Fat: 46.3%E Protein: 9.6%E	[71]
nondiabetic men with high T2D risk genotypes at the rs7901695 locus (*n* = 8): age: 31.2 ± 6.3 y, BMI (kg/m^2^): 28.5 ± 8.1) or Low T2D risk genotypes at the rs7901695 locus (*n* = 13): age: 35.2 ± 10.3 y, BMI: 28.1 ± 6.4	Plasma at 0, 0.5, 1 and 2 h after meal intake	UHPLC-Q-TOF		AUC from 0 to 120 min after challenge meal. In High Risk group compared to low risk group: Following High Carbohydrate meal: ↓ AUC phospholipids, lysophospholipids, sphingolipids, arachidonic and oleic acids, their metabolites: keto- and hydoxy-fatty acids, leukotrienes, uric acid and pyroglutamic acid. Following Normal Carbohydrate meal: ↑ AUCs of postprandial sphingosine Following both meal type: ↓ AUCs of acylcarnitines ↑ AUCs of fatty acid amides.	Mixed liquid meal Normal carbohydrate meal: Cubitan (Nutricia): 360 mL High carbohydrate meal: Nutridrink Juice Style, Fat Free, (Nutricia): 300 mL	450 kcal Normal carbohydrate meal: Carbohydrate: 45%E Lipid: 25%E Protein 30%E High carbohydrate meal: Carbohydrate: 89%E Lipid: 0%E Protein: 11%	[56]
Healthy men and women (*n* = 123) Mean and women with cardiometabolic disease (*n* = 226): Either obesity, diabetes, hypertension or metabolic syndrome	Plasma at 0 and 2 h after meal intake	LC-MS	In healthy participants: 1383 features were significantly changed In cardiometabolic disease group: 1756 features were significantly changed	22 metabolites differed after meal challenge and had different response depending on cardiometabolic disease status. Examples includes: - Acylcarnitines - Dipeptides (histidinyl-tryptophan or tryptophyl-histdine) - Phospholipids - Bile acid metabolites	Mixed liquid meal Incaparina (vegetable protein mixture): 12 g, skim milk (lactose-free): 170 mL, safflower oil: 25 g, sugar: 52 g	520 kcal Carbohydrate: 52%E Lipid: 42%E Protein: 6%E	[59]
Healthy men and women (*n* = 110) aged 18–40, divided in 4 groups: Normal healthy control (*n* = 30) 1st degree relatives of patients with T2D (*n* = 30) Overweight group (*n* = 30): BMI: 23–30 kg/m^2^ Prediabetes group (*n* = 20): Fasting glucose levels: 100–125 mg/dL	Plasma at 0, 1 and 2 h after meal intake	LC-QToF-MS		In Overweight group compared to control and T2D relative group: ↓ (fasting and fed state): MG(22:2(13Z,16Z)/0:0/0:0) and LPC (15:0) In prediabetes group compared to control group: ≠ LPE (0:0/18:2(9Z, 12Z)), LPE (0:0/20:4(5Z, 8Z,11Z,14Z)), 10, 11-dihydro-leukotriene B4 and 3-Oxocholic acid In men: Association with ↓ IS: triglycerides, VLDL, C-peptide, uric acid, xanthine and GCDC-3-glucuronide In women: Association with ↑ IS: HDL, leptin, adiponectin, glutathione-conjugate, phytosphingosine and lysophospholipids	Complex meal Idli, chutney (coconut and Bengal gram), milk tea, skimmed milk powder, table sugar and coconut oil	25% of the total daily energy required per day—calculated with ideal body weight Carbohydrate: 55% Lipid: 30% Protein: 15%	[69]
Three groups of men and women (45–65 years old) without any history of IFG or T2D stratified by fasting glucose concentrations: NGT (*n* = 176): Fasting glucose ≤ 6.0 mmol/L IFG (*n* = 186): Fasting glucose ≥ 6.1 and < 7.0 mmol/L T2D (*n* = 171)): Fasting glucose ≥ 7.0 mmol/L	Plasma 0 and 2.5 h after meal intake	FIA-ESI-MS/MS		NGT vs. T2D: At 150-min after challenge meal: a profile of four metabolites (acylcarnitine C16:1 and C4:1, glycine and LPC a C17:0) was able to distinguish the T2D group from the NGT group. Response profile (value at 150 min—value at fasted state): A profile of 16 metabolites (50% short-chain acylcarnitines) distinguished T2D and NGT groups.	Mixed liquid meal 400 mL Ingredients details not available	600 kcal Carbohydrate: 50%E Lipid: 34%E Protein: 16%E	[58]
Men aged 30–70 years old Healthy group (*n* = 20): BMI: 20.0–25.0 kg/m^2^ T2D group (*n* = 20): BMI: 25.1–34.9 kg/m^2^	plasma at 0, 0.5, 1, 2, 4, 6 and 8 h after meal intake	GC-MS	In healthy group: (selected examples) Late ↑: lipolysis (glycerol, several FFA) Late ↑: ketogenesis (3-hydroxybutanoicacid, acetoacetate, 2-hydroxybutanoic acid). ↑ followed ↓ to baseline: most AA ↑ followed ↓ to baseline: glycolysis (pyruvate and, glycerol-3-phosphate) ↓ followed ↑ to baseline: TCA cycle intermediates (succinate, malate and citrate)	T2D vs. healthy: 58 features different between groups: ↓ lipolysis (blunted glycerol and non-essential FFA response) ↓ ketogenesis (delayed ↓ and smaller ↑ for 3-hydroxybutanoic acid and acetoacetate) Higher ↑ for most AA (BCAA and derivatives, serine, lysine, threonine, glutamate and tyrosine)	High fat liquid meal 400 mL beverage, palm olein: 12.4% (weight/weight), dextrose: 17.25%, protifar (Nutricia): 4.13%, vanilla flavor: 0.10%, trisodium citrate: 0.12%, sodium hydroxide: 0.08%, water: 66.12%	950 kcal Carbohydrate: 33%E Lipid: 59%E Protein: 8%E	[63]

AA: Amino acids, AUC: Area under the curve, BMI: Body mass index, BCAA: Branched-chain amino acid, E: Energy, FFA: Free fatty acid, GCDC: glycochenodeoxycholic, HDL: High density lipoprotein, IFG: Impaired fasting glucose, IS: Insulin sensitivity, LPC: lysophosphatidylcholine, LPE: Lysophosphatidylethanolamine, MG: monoacylglycerol, NGT: Normal glucose tolerance, TCA: Tricarboxylic acid, T2D: Type 2 diabetes and VLDL: Very low density lipoprotein.

## Data Availability

Not applicable.
